# Prediction of bacterial E3 ubiquitin ligase effectors using reduced amino acid peptide fingerprinting

**DOI:** 10.7717/peerj.7055

**Published:** 2019-06-07

**Authors:** Jason E. McDermott, John R. Cort, Ernesto S. Nakayasu, Jonathan N. Pruneda, Christopher Overall, Joshua N. Adkins

**Affiliations:** 1 Biological Sciences Division, Pacific Northwest National Laboratory, Richland, WA, United States of America; 2 Department of Molecular Microbiology and Immunology, Oregon Health & Science University, Portland, OR, United States of America; 3 Center for Brain Immunology and Glia, University of Virginia, Charlottesville, United States of America

**Keywords:** Machine learning, Protein function, Sequence analysis, Virulence, Ubiquitination

## Abstract

**Background:**

Although pathogenic Gram-negative bacteria lack their own ubiquitination machinery, they have evolved or acquired virulence effectors that can manipulate the host ubiquitination process through structural and/or functional mimicry of host machinery. Many such effectors have been identified in a wide variety of bacterial pathogens that share little sequence similarity amongst themselves or with eukaryotic ubiquitin E3 ligases.

**Methods:**

To allow identification of novel bacterial E3 ubiquitin ligase effectors from protein sequences we have developed a machine learning approach, the SVM-based Identification and Evaluation of Virulence Effector Ubiquitin ligases (SIEVE-Ub). We extend the string kernel approach used previously to sequence classification by introducing reduced amino acid (RED) alphabet encoding for protein sequences.

**Results:**

We found that 14mer peptides with amino acids represented as simply either hydrophobic or hydrophilic provided the best models for discrimination of E3 ligases from other effector proteins with a receiver-operator characteristic area under the curve (AUC) of 0.90. When considering a subset of E3 ubiquitin ligase effectors that do not fall into known sequence based families we found that the AUC was 0.82, demonstrating the effectiveness of our method at identifying novel functional family members. Feature selection was used to identify a parsimonious set of 10 RED peptides that provided good discrimination, and these peptides were found to be located in functionally important regions of the proteins involved in E2 and host target protein binding. Our general approach enables construction of models based on other effector functions. We used SIEVE-Ub to predict nine potential novel E3 ligases from a large set of bacterial genomes. SIEVE-Ub is available for download at https://doi.org/10.6084/m9.figshare.7766984.v1 or https://github.com/biodataganache/SIEVE-Ub for the most current version.

## Introduction

Assignment of functional annotations for newly sequenced proteomes is accomplished largely through transference of annotations from existing proteins using sequence similarity. Many protein families exist that have shared sequence similarity and functional annotation and new members can be identified through established models such as hidden Markov models (HMMs). However, there are many other groups of proteins that have closely related functions but diverse sequences. These groups can be described with multiple models that capture different regions of sequence space but may include members that don’t have sequence similarity with other members detectable by traditional sequence methods.

Standard methods for developing sequence-based models such as HMMs rely on sequence alignment of family members as a first step. Models are then constructed using sequence signatures at specific locations established from those alignments. If sequence alignment is not possible or results in poorly aligned sequences, robust models for functionally related proteins may not exist. In these cases machine learning methods can be used to group proteins with similar function together based on sequence-derived features that do not require alignment. Such methods generally rely on general properties or biases (distributions of different amino acids across the entire protein, e.g.) or generating suites of short subsequences from the entire protein in a position agnostic approach. Such applications have developed models for problematic protein functions such as multidrug antibiotic resistance transporters (McDermott et al., 2015) by us, and DNA binding proteins (Qu et al., 2017), calmodulin-binding proteins (Abbasi et al., 2017) and to identify subcellular localization (Tung et al., 2017), immunogenic regions of proteins (Kuksa et al., 2015), and kinase specificity (Wang et al., 2017), by others. Our group previously developed a machine learning model to identify substrates of the bacterial type III secretion system, and this and similar models have been successful at identifying novel family members (Arnold et al., 2009; Samudrala, Heffron & McDermott, 2009; McDermott et al., 2011; Niemann et al., 2011; Hovis et al., 2013).

A versatile method for creation of subsequences for use in such models is the kmer approach, also known as string kernels. This method has been used in sequence analysis to identify distant homologs (Leslie, Eskin & Noble, 2002; Leslie et al., 2004), nucleotide-based functional features (Li & Jiang, 2005), and structural folds (Wang et al., 2017), and to predict antibody epitopes (Sher, Zhi & Zhang, 2017). A current limitation of this approach is computational. Since the alphabet used by amino acids is normally 20, the space of possible sequences of length k expands exponentially with k, rendering even short kmers of length 6 unwieldly since this requires representation of 20^6^ (64 million) possible features. Additionally, as kmers increase in length they become less common resulting in feature sets that are more distinct for each protein, and thus less likely to reveal underlying relationships. This problem can be addressed using mismatch kernels (Leslie et al., 2004) and similar approaches, but remains a computational and pragmatic barrier. Here we report the use of a kmer-based approach with the novel inclusion of a step which first reduces the alphabet used for representation to address these issues. We are able to explore much longer kmers, albeit with reduced information content, that can be used to represent general patterns in amino acid properties such as charge and hydrophobicity. We apply this approach to identification of novel ubiquitin E3 ligases in pathogenic bacteria.

Ubiquitination is an abundant protein post-translation modification (PTM) in eukaryotic cells that controls many key pathways, including protein turnover and innate immune signaling (Bhoj & Chen, 2009; Kravtsova-Ivantsiv & Ciechanover, 2012). Ubiquitination is a dynamic and reversible PTM produced by the coordinated action of three enzymes: E1 ubiquitin activating enzyme, E2 ubiquitin conjugating enzyme, and E3 ubiquitin ligase. The removal of ubiquitin units from proteins is catalyzed by deubiquitinating enzymes (Komander, Clague & Urbe, 2009; Metzger, Hristova & Weissman, 2012). Eukaryotic E3 ligases are mainly classified into two groups, HECT and RING, with different structural features and catalytic mechanisms. The first group is characterized by its HECT (homolog of E6-associated protein C-terminus) domain and during catalysis forms an intermediate that receives ubiquitin from the E2 conjugating enzyme before transferring to substrates (Metzger, Hristova & Weissman, 2012). The second type is characterized by the presence of a RING (Really Interesting New Gene) finger domain, which consists of a series of histidine and cysteine residues that coordinate binding to zinc ions. The RING-type E3 ligases do not form a ubiquitin-linked intermediate, but promote the direct ubiquitin transfer from the E2 to the targeted substrate (Metzger, Hristova & Weissman, 2012).

Although bacteria lack complete ubiquitination machinery, some pathogenic bacteria have evolved or acquired virulence effectors that can be introduced in to host cells via secretion systems and manipulate the process of ubiquitination through structural and/or functional mimicry (Rytkonen & Holden, 2007; Hicks & Galan, 2010). Although bacterial proteins that mimic the E1 and E2 enzymes have not been identified, a number of bacterial and viral E3 ligases have been shown to be enzymatically active and to be important for virulence (Rytkonen & Holden, 2007; Hicks & Galan, 2010). These E3 ligases expand the number of sequence families from eukaryotic ubiquitin ligases (Catic et al., 2007; Cui et al., 2010), with several displaying structural mimicry, i.e., similar structure and function arising from dissimilar sequence (Hicks & Galan, 2010). *E. coli* expresses a class of effector proteins named NleG-like proteins, after the first characterized member of this class, that contain U-boxes, a domain similar to RING but lacking the coordination with zinc ions, and were shown to be enzymatically active E3 ligases (Wu et al., 2010). Some Gram-negative bacteria have members of a class of E3 ligases named Novel E3 Ligases (NEL, not to be confused with NleG) that despite having a conserved cysteine residue at the catalytic site has little similarity to HECT domains (Singer et al., 2008). Members of NELs include virulence factors, such as *Shigella* IpaH and *Salmonella* SspH1, SspH2 and SlrP (Rohde et al., 2007; Singer et al., 2008; Bernal-Bayard & Ramos-Morales, 2009; Quezada et al., 2009; Levin et al., 2010).

Sequence family models have been developed as part of the popular Pfam database that can identify new members of the classes described above, but fail to identify E3 ligases that do not fall into these families. This lack of sequence similarity makes it difficult to characterize new ubiquitin ligase mimics in bacteria or viruses. While experimental techniques are essential to definitively characterize a protein’s function, they are time-consuming and expensive, making them unrealistic for genome-wide screening of effectors. Computational techniques are a better choice for identifying the putative function of uncharacterized proteins, which can later be verified by experimental assays. Since most protein structures have not been solved experimentally, computational techniques for identifying the function of uncharacterized protein rely upon the similarity of its amino acid sequence to that of a protein with a known function.

Here we present a novel method for alignment-free classification of proteins using kmers built from reduced amino acid alphabets. That is, physicochemical properties or other grouping strategies are used to group amino acids into sets that are then used to represent kmer feature sets. These feature sets are then used as input to an SVM using a family-wise cross-validation strategy and a classifying model is derived. Surprisingly, we found that an amino acid alphabet that represents residues as either generally hydrophobic or generally hydrophilic performed the best as features for classification yielding a classification receiver-operator characteristic (ROC) area under the curve (AUC) performance of 0.90, where an AUC of 0.5 corresponds to random chance and AUC of 1.0 is perfect classification of all positive and negative examples. Feature selection identified several regions of similarity across disparate families of E3 ubiquitin ligases. We predict a number of novel E3 ubiquitin ligases from a large set of genomes with this novel approach.

## Materials & Methods

### Dataset

We identified a set of 164 confirmed bacterial or viral E3 ubiquitin ligase effectors from the UniProt database by searching for ‘E3 ligase’ in manually annotated bacterial and viral sequences and manually checking the results for accuracy (Bairoch et al., 2005). Negative examples were 235 other bacterial effectors identified from literature (Lee, Mazmanian & Schneewind, 2001; Stebbins & Galan, 2001; Bairoch et al., 2005; Burstein et al., 2009; Quezada et al., 2009; Samudrala, Heffron & McDermott, 2009; Spallek, Robatzek & Gohre, 2009; Buchko et al., 2010; Collins & Brown, 2010; Hicks & Galan, 2010; Price & Kwaik, 2010; Komander, Clague & Urbe, 2009; Wu et al., 2010; Dean, 2011; Lin et al., 2011; McDermott et al., 2011; Anderson & Frank, 2012; Deslandes & Rivas, 2012; Xin et al., 2012). Though somewhat limited in size, this set of E3 ubiquitin ligase virulence effectors represents the state of current knowledge, and we have had success with similar approaches applied to smaller datasets previously (Samudrala, Heffron & McDermott, 2009; McDermott et al., 2015). We include details on the dataset as Supplemental Data.

To provide predictions for relevant bacterial pathogens we downloaded a set of 171 genomes that are listed as human pathogens and are representative reference genomes from PATRIC (Wattam et al., 2017). This set comprises 480,562 protein sequences excluding all of the proteins used in the training set above. A list of the genomes included in this study is provided in Supplemental Data.

### Features

Every protein sequence used for either learning or prediction is encoded by counting occurrences of peptides of varying length in the sequence in a manner similar to the previously described string kernel (Leslie, Eskin & Noble, 2002). The possible number of peptides greater than 4 amino acids long is very large (20^4^ = 160,000 peptides). We wanted to extend this approach to identify sequence patterns based on groupings of amino acids based on physiochemical or other properties. We therefore also encoded sequences to reduce the sequence space using one of several encodings (Table 1). Features were then generated for a range of different peptide lengths (3 to 20) and peptides that were observed in fewer than 10 examples were removed from consideration.

**Table 1:** Reduced amino acid (RED) encodings.

Name	Groups	Notes	Reference
NAT (Natural)	ACDEFGHIKLMNPQRSTVWY	No encoding	
RED1 (Hydrophobicity)	SFTNKYEQCWPHDR AGILMV	Hydrophilic Hydrophobic	Arnold et al. (2009)
RED2 (Physiochemical)	AGILMV PH FE NQST DE KR CY	Hydrophobic Hydrophilic Aromatic Polar Acidic Basic Ionizable	Arnold et al. (2009)
RED3 (Solvent accessibility)	CILMVFWY AGHST PDEKNQR	Low Medium High	Bacardit et al. (2009)
RED4 (Hydrophobicity and charge)	SFTNYQCWPH AGILMV KEDR	Hydrophobic Hydrophilic Charged	This study
RED5 (Hydrophobicity and structure)	SFTNKYEQCWHDR AILMV PG	Hydrophilic Hydrophobic Structural	This study

Features for each protein are generated by considering all peptides of length k in a sequence, encoding these (optionally) using the chosen encoding scheme, then counting the occurrences of the encoded peptide.

### Data partitioning

To remove bias created by having multiple examples with very similar features (i.e., closely related effectors from different organisms) we first partitioned the examples to identify clusters of related effectors. In order to achieve this partitioning, we clustered the sequences based on NCBI BLASTP similarity results. Parameters of BLASTP were set to their default values. Using a lower *E* value threshold (for example, *E* = 0) groups sequences more tightly and thus results in clusters that are likely to be more similar to another cluster and thus represent a generous division of families for the classification task using our cross-validation approach (see below). Conversely, higher *E* value thresholds (for example, *E* = 0.01) yield broader, more general clusters that are less likely to be similar to any other clusters, and thus represent a conservative division of families for our classification task. We used a more conservative threshold (E 0.01) to group the set of 407 proteins into 176 clusters of loosely related protein sequences. We examine the effect of varying the BLAST *E*-value threshold on the size of the generated protein families (Fig. S1). We next compared this approach to a more sophisticated approach for determining orthologous groups of proteins OrthoMCL, and found only minor differences with our approach resulting in joining three of our previous clusters (each containing a single protein each) in to one. We then used this final set of 174 clusters for our analyses.

### Cross validation

Cross validation (CV) is widely used to test the performance of a classification scheme on a given dataset. The entire dataset is partitioned into several non-overlapping folds. These folds are used as test sets. The corresponding training set for a particular fold consists of the remainder of the dataset. Each iteration of cross validation involves using a training set to generate a model and testing that model on the corresponding test set. This process is repeated until every fold has been tested.

The experimental setup of our study uses a variant of CV called Family-Wise Cross Validation (FWCV) to judge the performance of our classifier. FWCV places all the samples belonging to a particular cluster (see above) in a single test set, while the classifier is trained using the remaining data. This prevents model overfitting by reducing the trivial similarities between testing and training sets (i.e., those similarities based on traditional sequence similarity).

We use the following example to explain this process. In Table 2 protein sequences have been assigned a sequence family based on sequence similarity using traditional methods (like OrthoMCL). A FWCV run would select, for example, sequence families 1 and 4 to train on for a single fold. This would mean that sequences A, B, G, and H would be in the training set and sequences C, D, E, and F would be set aside for testing. A model would be trained on A, B, G, and H then applied to C, D, E, and F to assess performance. A good performance (as assessed by AUC) would mean that the information in sequences A and B from sequence family 1 could be used to predict the class of sequences C and D in sequence family 2. The process is then repeated for a number of folds (not useful for this limited example, but very useful in a real dataset), and the performance of the individual folds averaged to get an overall assessment of model performance.

**Table 2:** Cross-validation toy example.

**Protein sequence**	**Sequence family**	**Class**
A	1	positive
B	1	positive
C	2	positive
D	2	positive
E	3	negative
F	3	negative
G	4	negative
H	4	negative

### Classification

The Support Vector Machine (SVM) determines the optimally separating hyperplane between two sets of points in high-dimensional feature space each belonging to a different class (Noble, 2006). We utilized the radial kernel from the e1071 R library (version 1.6-8; Meyer et al., 2017) in our implementation.

The area under the curve (AUC) and receiver-operator characteristic curve (ROC) calculation was performed using the R library pROC (version 1.10.0; Robin et al., 2011).

### Feature selection

Feature selection was accomplished using two complementary methods. The first is the standard SVM Recursive Feature Extraction (SVM-RFE; algorithm as described in (Guyon et al., 2002)). We can obtain an ordering of the features using the absolute value of the entries of the SVM weight vector *w*. Each recursive feature elimination iteration involves eliminating the set of features that have the smallest absolute weight *w*_i_ until *k* features remain. The smaller and smaller sets of features obtained at each step represent predictive models that consider all the features together, but features selected vary due to the cross-validation approach we are using. In the second approach we established a simple metric to score individual features based on their representation in the positive example families versus negative example families. This was accomplished by calculating the score (S) for each feature (f) as the percentage of examples from each sequence cluster (M) that contained f, then calculating difference between the mean percentages for the set of positive families and negative families. Thus a positive score for a feature means that it is disproportionately represented in the positive examples while accounting for differences in the size of sequence families. Sets of individual predictive features were then used to train a minimal model for prediction as described in results.

### Implementation details and availability

Feature generation from sequences is performed using a standalone Python (version 3.6.3; (Foundation, 2018) script that uses the BioPython library (version 1.70; (Cock et al., 2009)). Training and validation of models was performed in R (version 3.3.3 (R Core Team, 2017). The SVM-RFE algorithm used by SIEVE-Ub was implemented in R as described by GIST-RFE (Guyon et al., 2002).

Code for the algorithm and datasets used to produce the results described in this paper are available at https://doi.org/10.6084/m9.figshare.7766984.v1 and the current version of SIEVEUb is available at https://github.com/biodataganache/SIEVE-Ub.

## Results

Known ubiquitin ligases fall into one of several sequence families, HECT, RING, and NEL, each of which can be identified using existing hidden Markov models (HMMs) from the Pfam database (PF00632, PF13639, PF14496). Additionally, sequence-based models exist for AvrPtoB (PF09046) and BRE1 (PF08647), which represent distinct E3 ubiquitin ligase families, and SopA (PF13981), which is a HECT-like domain. We analyzed the assembled sequences using the Pfam database and identified members of all these families (Supplemental Data). We note that, not surprisingly, each of these Pfam families map to a different sequence cluster identified by OrthoMCL, though NEL and RING are broken into more than one sequence cluster each. The family with the most representation in our set of positive examples is the NEL family with 102 members. Taken as a whole the nine Pfam models achieve an accuracy of 95% and a precision of 98% for prediction of E3 ubiquitin ligases from the background of other virulence effectors, with 14 known ubiquitin ligases being missed. It is important to note that neither the orthology approach we took to identify sequence clusters nor the individual Pfam models provided any predictive ability across sequence families. Our goal is to develop a generalized, alignment-free approach to predict members of this functional family capturing those not identifiable through a sequence-based model such as those in Pfam, and providing the potential to identify novel functional family members.

### Dissimilar ubiquitin ligases can be detected using reduced amino acid (RED) peptides

To provide feature sets that were specific enough to capture relationships between functionally similar proteins, yet general enough to identify regions of similarity between divergent sequences we adapted the kmer approach to represent protein sequences by a series of all peptides of length k from that sequence. Our novel extension translates each amino acid in the sequence to a smaller number of groups based on physicochemical properties or other arbitrary grouping methods- a reduced amino acid (RED) alphabet. Initially we chose three reduction mappings based on previously reported approaches: hydrophobicity (RED0), standard physiochemical properties (RED1), and solvent accessibility (RED2) (Arnold et al., 2009; Bacardit et al., 2009). The groups are listed in Table 1.

The set of positive and negative examples for E3 ubiquitin ligases was encoded using each of the REDs and the native sequence, and peptide kmers of various lengths were counted for each. Peptides present in fewer than 10 examples were excluded from further consideration. Each dataset was then split into independent training and testing sets on a sequence cluster-wise basis (that is, clusters of similar sequences as determined by OrthoMCL were kept together in the training or testing set), based on a conservative cluster grouping (see methods). Cluster-wise splits and associated training and testing were performed 100 times for each model and the score (SVM discriminant) for each example averaged. Average scores were used to determine ROC AUC for each model and results are presented in Table 3 and Fig. S1. We were concerned that the cross-validation procedure might be affected by the large size of some of the sequence families (an NEL cluster has close to 100 members) or by how the families were chosen during cross-validation. We examined the first problem by reducing the size of the large families to a maximum value and rerunning the analysis, finding that this did not affect the results. Similarly, choosing different random seeds prior to cross-validation did not significantly affect results (see Supplemental Data).

**Table 3:** Best model performance.

****	**Kmer length**	**AUC**
NAT	17	0.851
RED0	14	0.903
RED1	6	0.803
RED2	8	0.742
RED3	6	0.884
RED4	13	0.814

Surprisingly, the models using RED0, a simple division of amino acids into hydrophobic and hydrophilic residues, performed the best for nearly all peptide lengths with a maximum AUC of about 0.85. The maximum AUC observed occurs with RED0 and a peptide length of 14 (RED0-K14) and so we focused on characterization of this model for the remainder of the article. Our results indicate that a simple encoding of amino acids can be used to classify effectors with E3 ubiquitin ligase function from other effectors, and from other non-effector proteins in general (see Prediction of novel E3 ubiquitin ligase mimics, below), with good confidence.

**Figure 1:**
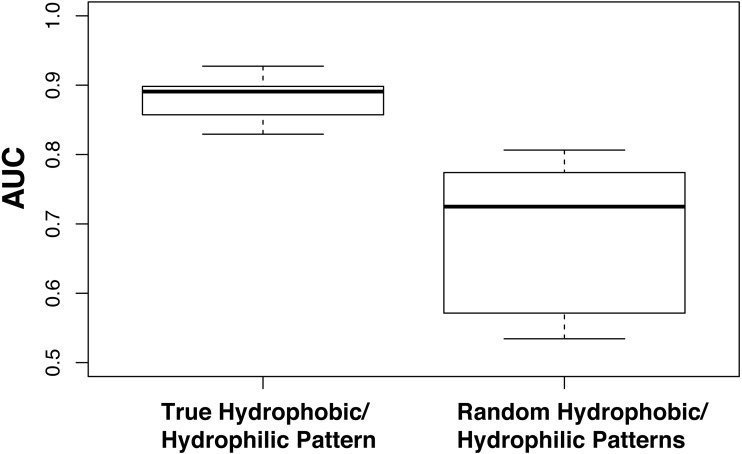
Amino acid reduction based on physicochemical properties is important. Models were evaluated using the standard hydrophobic/hydrophilic reduction alphabet (RED0) and randomly divided sets of amino acids (RND0) with a kmer length of 14. Performance was evaluated using 100 fold family-wise cross validation and AUC. The plot shows that a division of amino acids into hydrophobic and hydrophilic residues outperforms a random division of amino acids.

We hypothesized that the performance of the RED0 is based on accurately representing the pattern of hydrophobic and hydrophilic residues in kmers. To examine this hypothesis we applied a family-wise cross-validation approach using ten alphabets where residues had been randomly assigned to either the hydrophobic or hydrophilic groups preserving the overall balance of hydrophobic to hydrophilic residues in the resulting random alphabet (6:14; see Table 1). We compared the performance of these random binary REDs at a kmer size of 14 with the true hydrophobic/hydrophilic RED0-K14 also run ten times to show the variability in partitioning of training and testing sets inherent in our approach and show the results in Fig. 1. In all cases the true RED0 outperforms the randomized REDs supporting our hypothesis though we note that there is a wide range of performances given with random binary REDs. We believe this is due to some random assortments containing reasonable divisions of residues between hydrophobic and hydrophilic residues because of the very simple nature of this division.

### SIEVE-Ub identifies biologically functional peptides

To identify a minimal set of features that are important for classification of E3 ubiquitin ligases from other effectors we used recursive feature elimination, a standard machine learning approach (Samudrala, Heffron & McDermott, 2009). Briefly, a model is trained on all features using our family-wise cross-validation approach, then weights for each feature are used to discard 50% of the features with the lowest impact on model performance. The remaining features are then used in another model training round in which this process is repeated until all the features have been eliminated. Using this approach we found that performance of the model dropped off when the number of features was still quite large, >2,000, and so does not identify a minimal set of features important for discrimination of examples. The training performance results from the RFE on the RED0-K14 model are shown in Fig. S2.

**Figure 2:**
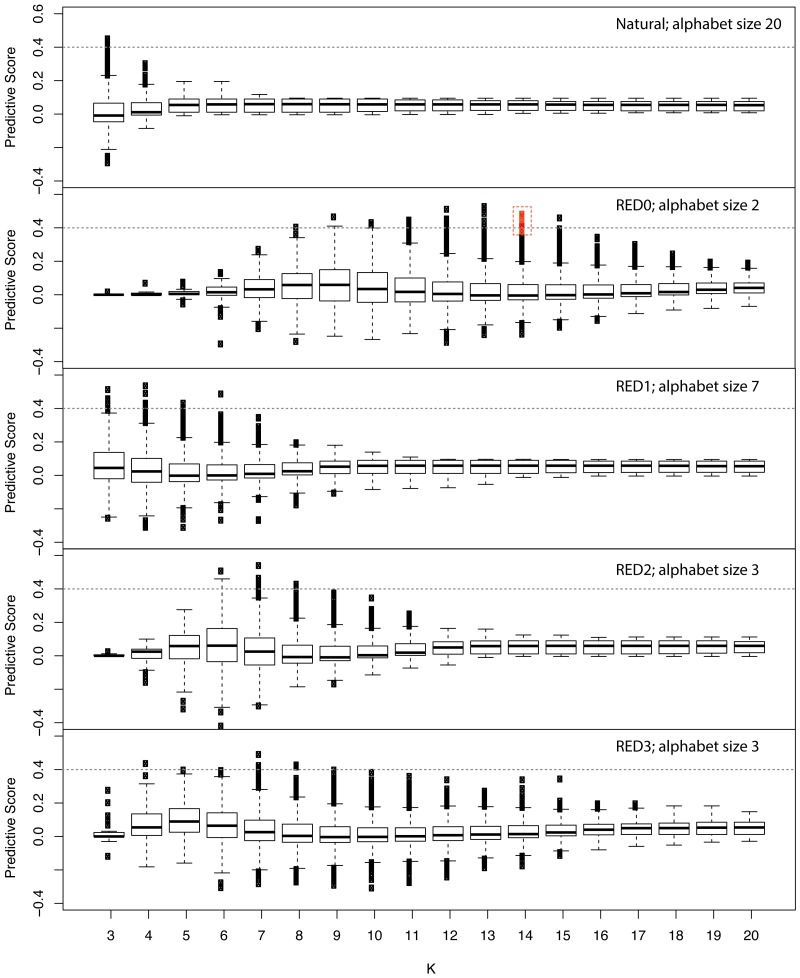
Assessing the information content of reduced amino acid kmers for ubiquitin ligase prediction. Family-normalized counts for kmer occurrence in positive and negative examples from the ubiquitin ligase examples used in the study were calculated and a differential score derived where 1.0 signifies kmers that are absolutely conserved in every example from the known ubiquitin ligase examples and not present in the negative examples and 0 is neutral in terms of representation. The different amino acid encodings are shown in each panel with the length of the kmer used indicated on the *X* axis and the box and whiskers representing the overall distribution of scores for all observed kmers. The red box indicates the minimal 10 kmer model described in the text. This plot shows that the simple hydrophobic/hydrophilic encoding (RED0) displays the greatest flexibility for the longest kmer lengths when predicting this class of proteins.

Since RFE failed to identify a minimal set of predictive features we developed a simple scoring metric to evaluate each feature independently to identify those features with disproportionate representation in the positive example set, while accounting for differences in sequence family sizes (see Methods). We applied this score across various kmer lengths and REDs and show the results in Fig. 2. Similar to the results we obtained in performance of models incorporating all features (Fig. S2), this approach shows that RED0 results in the longest kmers that are specific to positive examples with lengths of 12–14. This also shows that the naturally occurring amino acid sequence does not produce kmers specific to the positive examples, highlighting the strength in using our RED approach.

We trained models using our family-wise cross-validation approach with the top most predictive kmer features from RED0 with a kmer length of 14 for consistency with our previous results, and found that the most predictive model performed quite well (AUC 0.87) with just ten features. The features from the minimal model are provided as Supplemental Data along with their locations in each of the positive and negative examples in our analysis set.

**Figure 3:**
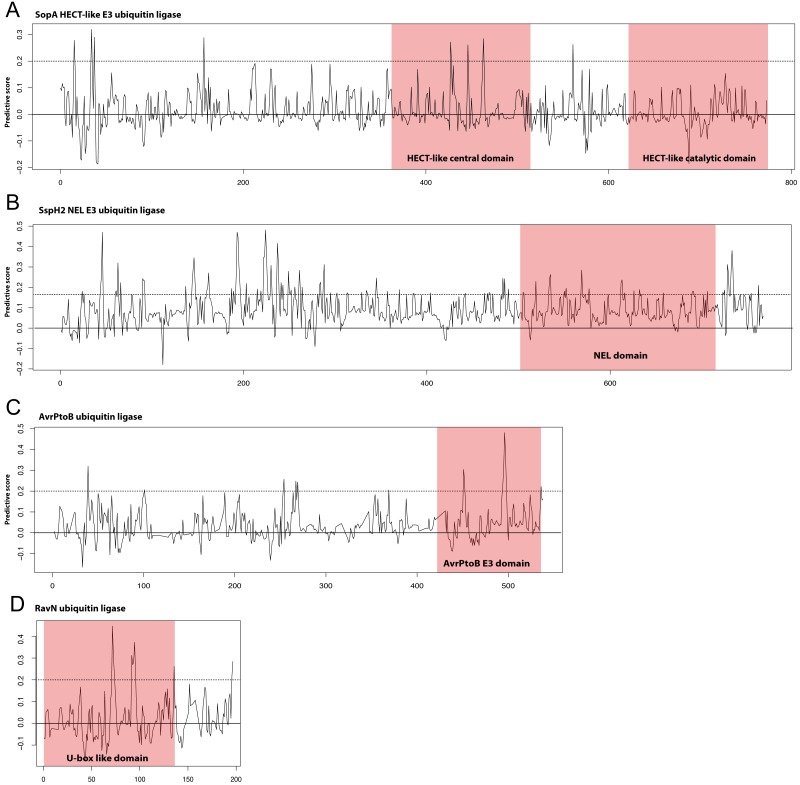
Discriminating peptides in E3 ligase domains. Differential scores were calculated for each position in the example E3 ligases shown that represent how unique the kmer at that location is across all known ubiquitin ligase examples used in the study. Examples shown are (A) the HECT-like Salmonella Typhimurium SopA, (B) the NEL family Salmonella Typhimurium SspH2, (C) the Pseudomonas syringae AvrPtoB, and (D) the recently discovered Legionella pneumophila RavN. This score was normalized for sequence families and a score of 1.0 represents a position that is completely conserved in the positive examples and not present in the negative examples. Kmers with scores of greater than 0.2 (dotted line) are significantly predictive of the functional class. Known E3 ligase domains are indicated in the shaded boxes. The RavN protein is a recently discovered E3 ubiquitin ligase with no sequence similarity with any existing examples and was not included in our training set. Combined with the ability of SIEVEUb to accurately predict ubiquitin ligase function these plots collectively indicate that some of the most predictive kmers are present in the known domains, despite the family-wise cross-validation approach that was used to prevent trivial sequence similarity inside families from impacting the results.

Though the E3 ligase examples used as our positive examples are diverse in terms of sequence many do fall into the families of E3 ligases described in the Introduction; HECT/U-box, RING, and NEL. We hypothesized that the most predictive kmers identified in our analysis would map to the known E3 ligase domains in these proteins. We show in Fig. 3 our scoring metric across four examples from different E3 ligase families. Significantly predictive peaks corresponding to highly predictive kmers can be found in each of the known domains from the HECT-like, NEL, AvrPtoB, and recently identified RavN effectors demonstrating that our approach is able to establish connections across disparate sequence families. We note that, except in the case of AvrPtoB, the most predictive kmers map to regions outside the known E3 ligase domains, indicating the presence of other signals that might be important in prediction.

We also applied our model to the recently discovered E3 ubiquitin ligase effector RavN from Legionella, which was not included in our training set (Lin et al., 2018). Our method predicted RavN to be an E3 ubiquitin ligase with a probability of 85%, despite having no detectable sequence similarity with other known E3 ligases. Though this is a limited validation it demonstrates the power of our approach at identification of novel E3 ligase effectors that have vastly divergent sequence.

### Prediction of novel E3 ubiquitin ligase mimics

To predict novel E3 ubiquitin ligase mimics in a larger set of sequences we applied the model described above (kmer 14 in RAA0, top 2000 most important features) to a set of over 400,000 proteins from representative human pathogens obtained from the PATRIC database (Wattam et al., 2017). We found only 67 proteins with positive SIEVEUb scores greater than 0.5, indicating that potential ubiquitin ligases are not common as assessed by our approach. We note that PATRIC annotations include E3 ubiquitin ligase functions, and none are detected in the subset we’ve focused on. This indicates that the predictions we’ve made are truly novel. We further filtered this list to include predictions that occurred in bacteria containing type III, IV, or VI secretion systems. This yields a list (Table 4) of predicted E3 ubiquitin ligases that are in organisms capable of delivering effector proteins in to the eukaryotic host cell cytoplasm, though we note that such effectors could be secreted via other mechanisms. Several of these predictions are annotated as enzymes, which could be false positive predictions. However, many virulence effectors are known to be multifunctional and annotation of functions is prone to error.

**Table 4:** Proteins predicted to be similar to ubiquitin ligase mimic set. *annotation based on sequence comparison only.

			**Secretion potential**				
**ID**	**Genome name**	**SIEVEUb Score**	**III**	**IV**	**VI**	**Gene name**	**Description**
APZ00_07775	*Pannonibacter phragmitetus* strain 31801	0.62	0	8	0		2-methylfumaryl-CoA hydratase
KKKWG1_2059	*Kingella kingae* strain KWG1	0.61	0	15	0		UPF0758 family protein
LV28_06870	*Pandoraea pnomenusa* strain DSM-16536	0.60	17	0	0		Benzaldehyde dehydrogenase
AB185_15825	*Klebsiella oxytoca* strain CAV1374	0.58	0	5	0		N-acetyltransferase ElaA
PMI0843	*Proteus mirabilis* HI4320	0.57	6	4	1		Low-affinity putrescine importer PlaP
NC_006155	*Yersinia pseudotuberculosis* IP 32953	0.53	63	8	2		hypothetical protein
LV28_00130	*Pandoraea pnomenusa* strain DSM-16536	0.53	17	0	0		MBL-fold metallo-hydrolase superfamily
APZ00_04010	*Pannonibacter phragmitetus* strain 31801	0.52	0	8	0		Soluble lytic murein transglycosylase
APH_0317	*Anaplasma phagocytophilum* HZ	0.51	0	24	0	fabH	3-oxoacyl-[acyl-carrier-protein] synthase

## Discussion

We note that the intent of our study was to develop a model that could identify E3 ubiquitin ligases based on protein sequence with reasonable accuracy and precision, which we demonstrated clearly. As such, we did not fully explore the range of possible parameters such as choice of SVM kernel, or other machine learning approaches that would work on our input features, to determine an optimal model. Our results show that we can use models based on highly divergent sequences to robustly predict E3 ubiquitin ligase function in bacterial and viral effectors. It is unclear how many E3 ubiquitin ligases that may exist but have not yet been discovered, and this question will only be answered through experimental validation of predictions made by our method, similar to the validation we have done for the original SIEVE (Samudrala, Heffron & McDermott, 2009).

## Conclusions

The general approach we describe, using peptides with reduced amino acid alphabets as features for machine learning, could be easily applied to other problems of functional classification given appropriate positive and negative example sets. We show that this approach can be used to discriminate effectors with E3 ubiquitin ligase activity from other effectors with good confidence and present a single model that is able to identify E3 ubiquitin ligases from different sequence families. Importantly, development of this model does not require sequence alignment of any kind. From this analysis we have presented an example of this approach identifying functionally important regions with dissimilar sequences, but similar structures. However, further work is necessary to explore the possibility that this is a more general property of the approach. This is the first algorithm dedicated to prediction of E3 ligase function in non-eukaryotic proteins.

##  Supplemental Information

10.7717/peerj.7055/supp-1 Figure S1 Performance of reduced amino acid (RAA) models on Ub ligase family-wise classification Models were generated for various lengths of peptide (*X* axis) using different RAAs (see Table 1) as described in the text. Training and testing were performed on independent sets 100 times and the average scores for each example used to calculate ROC AUCs. The plot shows that a simple grouping of amino acids by general hydrophobicity provides the best performance.

10.7717/peerj.7055/supp-2 Figure S2 Recursive feature elimination for selection of features important in the *k* = 14 and hydrophobic/hydrophilic RED0 alphabet Recursive feature elimination (RFE) with family-wise cross-validation was applied to all examples using 14mers and the RAA0 and AUC assessed for each model. The plot shows that good performance can be achieved with >1,000 features but that performance falls off with fewer features. We note that a simple scoring metric is able to identify a minimal subset of features that retains good predictive performance, pointing out the limitation of the RFE procedure in this particular case.

## Additional Information and Declarations

### Data Availability

The following information was supplied regarding data availability: Data is available at GitHub: https://github.com/biodataganache/SIEVE-Ub.
