# Supplementary figures and images for: Prediction of bacterial E3 ubiquitin ligase effectors using reduced amino acid peptide fingerprinting
Source: PeerJ. 2019 Jun 7;7:e7055. doi: 10.7717/peerj.7055 (PMC6557245; doi:10.7717/peerj.7055)

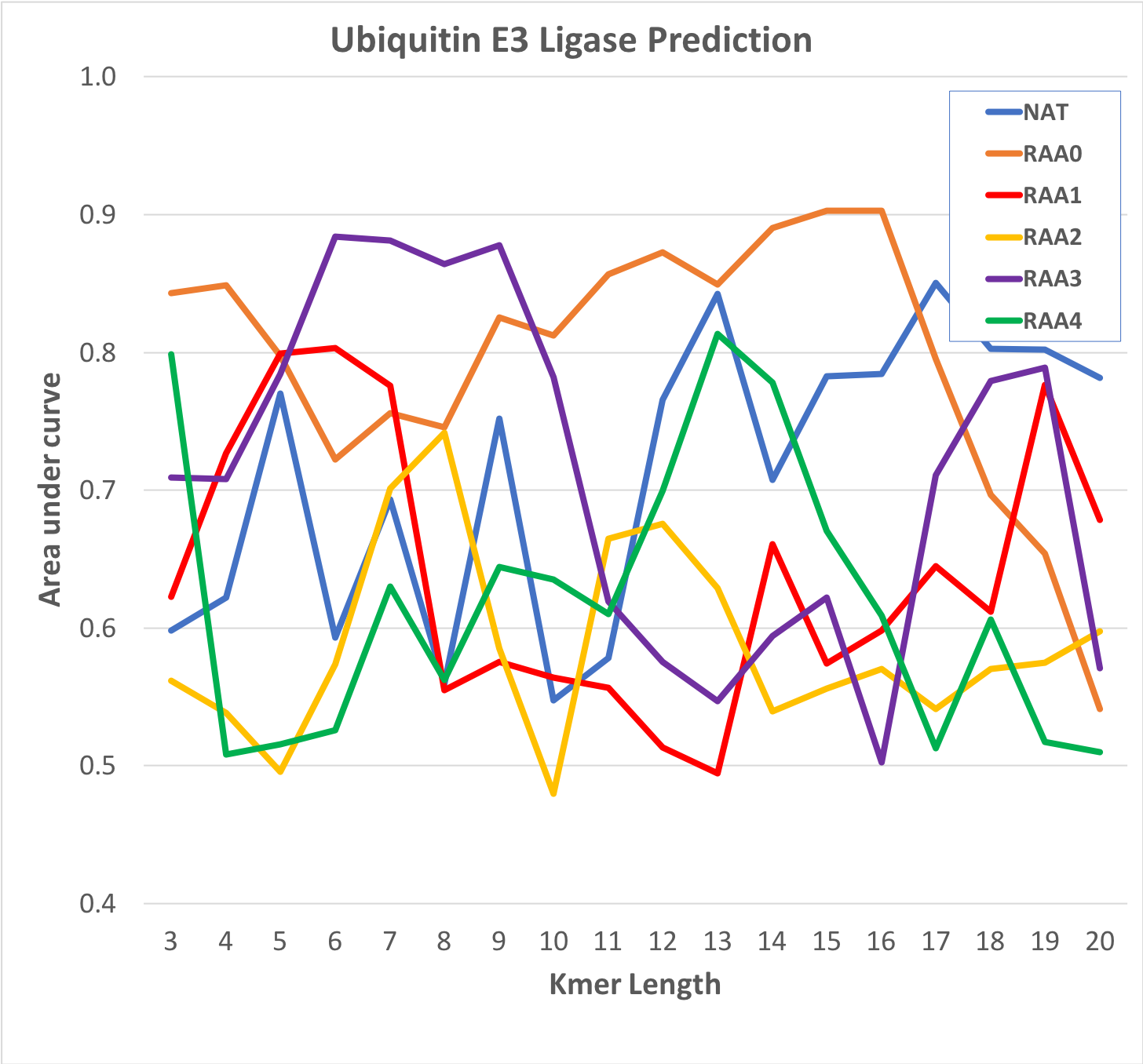

Supplement: Figure S1 — Models were generated for various lengths of peptide (X axis) using different RAAs (see Table 1) as described in the text. Training and testing were performed on independent sets 100 times and the average scores for each example used to calculate ROC AUCs. The plot shows that a simple grouping of amino acids by general hydrophobicity provides the best performance. [file peerj-07-7055-s001.png]

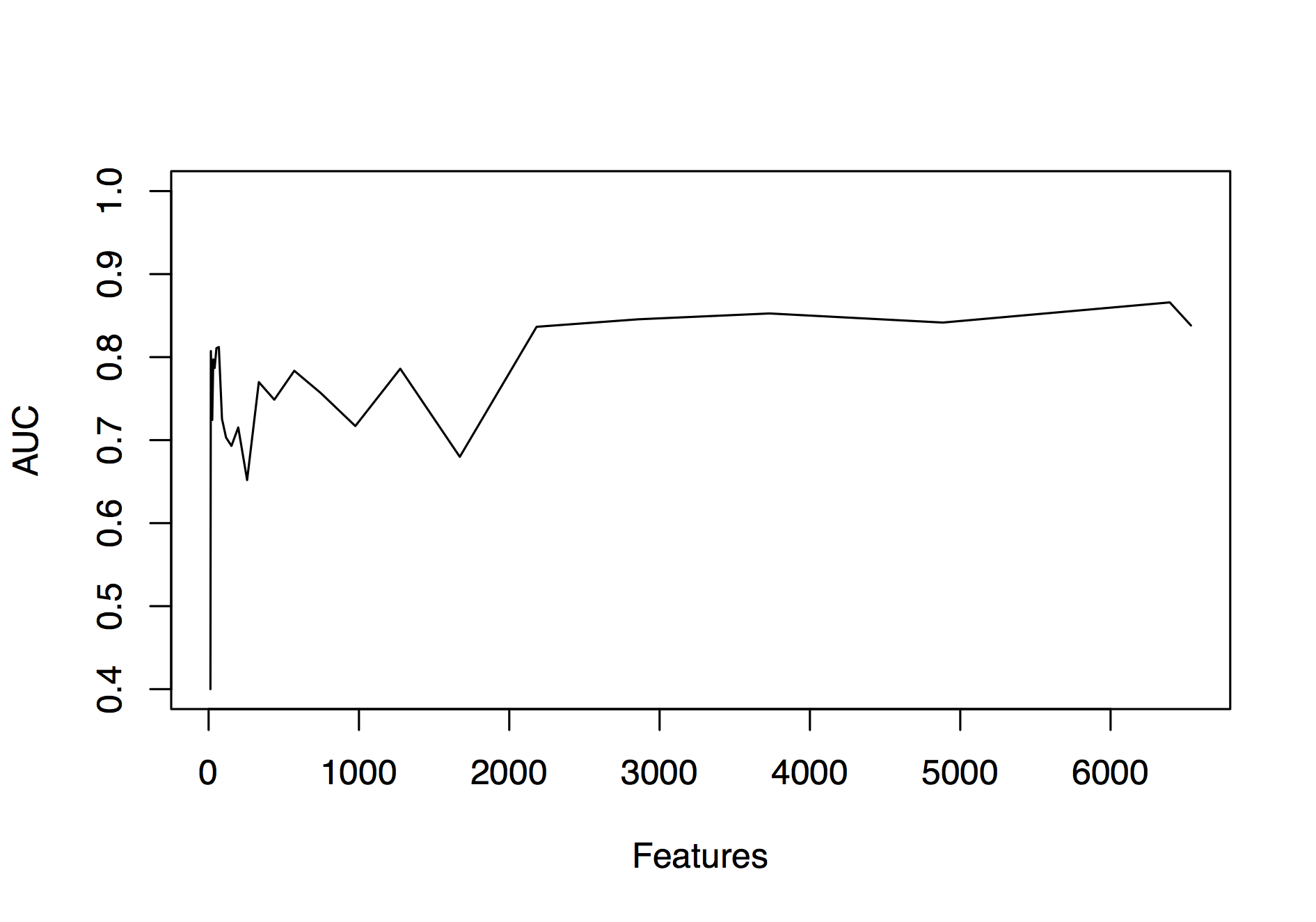

Supplement: Figure S2 — Recursive feature elimination (RFE) with family-wise cross-validation was applied to all examples using 14mers and the RAA0 and AUC assessed for each model. The plot shows that good performance can be achieved with >1,000 features but that performance falls off with fewer features. We note that a simple scoring metric is able to identify a minimal subset of features that retains good predictive performance, pointing out the limitation of the RFE procedure in this particular case. [file peerj-07-7055-s002.png]
